# Cyclooxygenase-dependent contribution to increased flow mediated dilatation following a week of repeated ischemic preconditioning

**DOI:** 10.1007/s00421-026-06324-4

**Published:** 2026-06-29

**Authors:** Samuel Thomas, Sophia R. Murphy, Sultan E. AlSalahi, Rehan T. Junejo

**Affiliations:** 1 Department of Life Sciences, Faculty of Science and Engineering, Manchester Metropolitan University, Chester Street, Manchester, M1 5GD UK; 2 Liverpool Centre for Cardiovascular Science, University of Liverpool and Liverpool Heart and Chest Hospital, Liverpool, UK

**Keywords:** Cyclooxygenase, Endothelium, Flow mediated dilation, Ischemic preconditioning, Prostacyclin, Remote ischemic preconditioning

## Abstract

**Purpose:**

Ischemic preconditioning (IPC) and remote ischemic preconditioning (RIPC) independently enhance endothelial function and flow mediated dilation (FMD); yet the underlying mechanisms remain incompletely defined. This study investigated the contribution of cyclooxygenase (COX) dependent vasodilators to FMD before and after repeated IPC and RIPC.

**Methods:**

21 healthy participants underwent Radial artery FMD assessments before and after oral aspirin (600 mg) ingestion for COX inhibition. Participants then completed a one-weeklong alternate day unilateral IPC protocol (four repeated cycles of 5-minute ischemia and 5-minute reperfusion). 24 h after the final IPC session, FMD responses were reassessed before and after aspirin ingestion.

**Results:**

Prior to IPC treatment, aspirin failed to significantly reduce FMD in both Radial arteries. Following IPC treatment, the now significantly increased FMD of the ipsilateral arm (9.50 ± 4.58% (mean ± standard deviation); *p* = 0.006) attenuated with aspirin (4.55 ± 2.68%; *p* = 0.002). Absolute difference in FMD % before and after aspirin ingestion increased from 1.48 ± 5.05% pre-IPC to 4.95 ± 4.96% (*p* = 0.016) post-IPC. In the contralateral (RIPC) arm, increased FMD % also attenuated with aspirin (*p* = 0.006) but this statistical significance was not seen following correction for shear rate. Nonetheless, absolute difference in FMD % before and after aspirin increased from 0.04 ± 4.05 pre-RIPC to 2.27 ± 3.60% (*p* = 0.043) post-RIPC.

**Conclusion:**

One week of alternate day IPC improves contribution of COX-derived vasoactive mediators to FMD. Contributions to RIPC driven improvements in FMD require further investigation.

**Supplementary Information:**

The online version contains supplementary material available at https://doi.org/10.1007/s00421-026-06324-4.

## Introduction

The vascular endothelium plays a central role in maintaining cardiovascular health including regulation of vasomotor tone, inflammation, coagulation, and vascular permeability (Sandoo et al. 2010). Endothelial dysfunction, characterized by a diminished capacity of endothelial cells to produce vasodilatory mediators like nitric oxide (NO) and prostacyclin (PGI_2_) along with increased propensity for platelets to aggregate, is a hallmark of early vascular disease and a strong predictor of future adverse cardiovascular outcomes (Rajendran et al. 2013). Strategies that preserve or restore endothelial function are therefore of considerable interest.

Ischemic preconditioning (IPC), a technique involving the application of brief, repeated cycles of ischemia and reperfusion, has shown promise in exerting protective effects across the cardiovascular system. IPC was initially developed in the context of cardiac protection where it delays cardiomyocyte injury (Murry et al. 1986) and reduces myocardial and vascular damage during ischemic events (Eisen et al. 2004). One of the most promising physiological benefits of IPC is its capacity to improve endothelial function. Notably, the protective effects of IPC are not limited to the site of ischemic application. Mounting evidence also supports the existence of remote IPC (RIPC) where vascular and tissue protection occurs in regions that are not directly exposed to the ischemic stimulus (Loukogeorgakis et al. 2005; Kharbanda et al. 2002).

Flow-mediated dilation (FMD), attributed to shear mediated production of endothelial NO (Green et al. 2014), is a marker of conduit artery endothelial health. FMD acutely increases following a single IPC / RIPC session (Moro et al. 2011). Repeated exposure elicits sustained improvements (Jones et al. 2014). These benefits have not only been demonstrated in healthy individuals (Jones et al. 2014; Bond et al. 2024), but also in clinical populations (Manchurov et al. 2014; Moro et al. 2011), highlighting IPC’s / RIPC’s potential utility as a non-invasive intervention for enhancing endothelial health in both preventative and therapeutic settings.

The mechanisms underlying the vascular benefits of IPC are however not yet fully understood. A commonly proposed explanation is that IPC enhances local and remote endothelial function through increased production and/or availability of endothelial NO (Rifkind et al. 2014; Chen et al. 2002). However, besides NO (Cyr et al. 2022), cyclooxygenase (COX) derived products such as PGI_2_ also contribute to endothelial health (Schror 1985). For example, use of *N*^*G*^-monomethyl-L-arginine (L-NMMA) attenuates but does not completely abolish Radial artery FMD (Joannides et al. 1995; Pyke et al. 2010), suggesting there may be more to FMD response than just endothelial NO. Whilst evidence of substantial prostanoid contribution to FMD of conduit arteries is equivocal (Joannides et al. 1995; Parker et al. 2011), endothelial cells are known to produce PGI_2_ under elevated shear stress (Russell-Puleri et al. 2017; Okahara et al. 1998). However, whether repeated IPC and/or RIPC driven improvements in endothelial health translate into an enhanced contribution of COX derived endothelial vasodilator prostanoids to conduit artery FMD response remains unclear.

Herein, we investigate the contribution of COX derived vasodilator prostanoids on shear stress mediated endothelium dependent FMD observed locally and remotely in healthy young individuals. We hypothesized that the vasodilator prostanoids would be involved in the enhanced FMD response observed with repeated IPC / RIPC.

## Methods

### Ethical approval

Ethical approval for the study was granted by the Science and Engineering Research Ethics and Governance Committee at Manchester Metropolitan University (64174). All procedures adhered to the established ethical standards and were conducted in accordance with the Declaration of Helsinki (2008). All potential participants received verbal and written information detailing the study procedures. Participants were given the opportunity to ask questions and receive clarification before providing written informed consent.

### Participant characteristics

A total of 21 participants (*males* 13 / *females* 8; Table 1) were recruited from the Manchester Metropolitan University and surrounding areas. Six participants (*males* 4 / *females* 2) completed the FMD assessments of both IPC and RIPC arms. Further 5 (*males* 3 / *females* 2) only participated in the IPC assessments (i.e., group total 11) and 10 (*males* 6 / *females* 4) only participated in RIPC assessments (i.e., group total 16). All participants were in good health, non-vapers / non-smokers, and not taking any medications (Table 1). Exclusion criteria included known allergies to aspirin or salicylates, use of non-steroidal anti-inflammatory drugs (NSAIDs), excessive weekly alcohol consumption, pregnancy, and use of any hormone-based contraceptives. Participants were also required to abstain from over-the-counter supplements / medications for at least five days prior to the initial examination and then for the duration of the study. None of the participants reported use of any over-the-counter medications. First visit (day 1 of experimental testing; please see below) for female participants was carried out within the early follicular phase of the menstrual cycle (≤ day 4 of menstrual cycle).

**Table 1:** Characteristics of all study participants

Characteristics	
Age, years	24 [22–30]
Sex, *m*/*f*	13/8
Height, cm	174 ± 9
Weight, Kg	81 ± 20
BMI, Kg/m^2^	25 [24–29]
Systolic blood pressure, mmHg	118 ± 11
Diastolic blood pressure, mmHg	76 ± 8
Mean arterial blood pressure, mmHg	93 ± 8
Pulse pressure, mmHg	42 ± 11
Alcohol use (*n*)	8
Multivitamins use (*n*)	6
Iron use (*n*)	1
Fish Oil Use (*n*)	2

Descriptive characteristics of the participants in the study (*n* = 21). Continuous variables are presented as mean ± SD for normal distributed data and median [IQR] for non-normal distributed data. Discrete quantitative data are presented as count

*BMI* body mass index

### Experimental protocol

Participants attended the temperature-controlled laboratory on five occasions over an eight-day period. Radial artery FMD assessments were carried out before and after COX inhibition on days 1 and 8 only. On FMD data collection days, participants were instructed to avoid vigorous exercise for 24 h, abstain from caffeine, fruit juices, and smoothies for 12 h, and fast for at least 6 h before arrival. On the first visit (day 1), participants provided written informed consent, completed health questionnaires, and underwent anthropometric measurements. Height, weight, waist circumference (at the level of the umbilicus) and hip circumference (at the level of the greater trochanter) were recorded. Following 10 min of supine quiet rest, Brachial artery blood pressure (BP) and heart rate (HR) were measured three times at 30-second intervals with an automated oscillometric monitor (M6, Omron, Kyoto, Japan) and averaged.

For FMD assessment, a pneumatic occlusion cuff (Hokanson, Bellevue, WA, USA) was placed around the wrist whilst the arms lay supported at the heart level. Both Radial arteries were insonated at 60° using Duplex-Doppler ultrasound (Terason uSmart 3300, Teratech Corporation, Burlington, USA) with 4–15 MHz multifrequency linear-array transducer (15L4A, Teratech Corporation, Burlington, USA) to capture vessel diameter and flow velocity. Ultrasound probe was held in place using a commercially available adjustable probe holder. Once an optimal image was obtained, the FMD protocol commenced with 1 min of baseline imaging, followed by 5 min of wrist cuff occlusion at supra-systolic pressure (> 240 mmHg), and a 3-minute post-cuff-deflation recovery period. Following initial FMD assessments under no/pre-aspirin condition, participants orally ingested 600 mg of over-the-counter soluble aspirin (acetylsalicylic acid). After a 30-minute period of quiet rest, FMD responses were reassessed. The dose and duration of aspirin used have previously been shown to successfully inhibit COX and platelet activity (Heavey et al. 1985; Hobl et al. 2015). Moreover, soluble preparations of aspirin as used here rapidly absorb and peak plasma concentrations of acetylsalicylic acid are reached within ~ 30-minutes (Benedek et al. 1995; Hobl et al. 2015; Gatti et al. 1989). Following these initial FMD assessments on day 1, participants then completed the weeklong unilateral IPC protocol (described below). On day 8, FMD assessments were repeated on the intervention (IPC-treated) or contralateral RIPC (control) arms as previously described. All Radial artery ultrasound video files were screen captured and stored for later offline analysis with an automated vessel-wall-tracking software (Cardiovascular Suite, version 3.4.1; FMD Studio, Pisa, Italy). Electrocardiogram (ECG) gating was not performed as cardiovascular Suite filters out the pulsatile component of cardiac-cycle to yield a continuous second-by-second vascular diameter data rather than a spot measurement of peak diameter. This approach is consistent with the current expert consensus technical recommendations on FMD (Thijssen et al. 2019) and has previously been used (Khan et al. 2021; Junejo et al. 2024; Thomas et al. 2025). To minimise the influence of circadian variations on endothelial function, all FMD measurements (before and after IPC treatment) were conducted either in the mornings or early afternoons; choice of the time interval was dependent on participant availability but remained consistent for each participant.

### IPC procedure

Participants were required to undergo a one week of unilateral IPC intervention on alternative days (i.e., days 1, 3, 5, and 7) beginning on day 1 of the study following FMD assessments. An aneroid sphygmomanometer’s cuff was wrapped around the upper arm of non-dominant hand. IPC protocol consisted of 4 repetitive cycles of blood pressure cuff inflations (> 220 mmHg) for 5 min followed by 5 min of complete cuff deflation. To ensure compliance with the protocol, sessions were completed in the presence of a researcher. For IPC session on the weekend, if necessary, participants were trained on how to perform the IPC procedure and provided with the equipment to undertake this at their homes; video evidence of successful completion of IPC procedure was sought to ensure protocol compliance.

### Data analysis

Body mass index was calculated as a ratio of weight-to-height squared. Pulse pressure was determined as systolic BP (mmHg) − diastolic BP (mmHg). Mean BP was determined as diastolic BP (mmHg) + [0.33 + (HR × 0.0012)] × [pulse pressure (mmHg)] (Razminia et al. 2004). For FMD assessment, data from the 1-minute baseline period were averaged. The peak Radial artery diameter following cuff deflation, relative to the average baseline diameter was defined as FMD and expressed as an absolute (mm) and relative (%) change. Shear rate (SR) was calculated as a ratio between arterial flow velocity x 4 and the arterial diameter. Shear rate area under the curve (SR_AUC_) was measured as an integral between cuff deflation and peak artery dilation. To determine the impact of COX inhibition, delta (∆) change in FMD % was calculated as the difference between pre-aspirin and post-aspirin ingestion FMD % for both IPC and RIPC arms. To determine the impact of SR_AUC_ on FMD data, SR_AUC_-corrected FMD % (FMD_SRAUC_) was determined by implementation of SR_AUC_ as a covariate in the analysis. In order to ensure compliance with the baseline diameter allometric scaling guidelines (Atkinson et al. 2013), the upper bound 95% confidence intervals (CIs) and slope of the log-transformed baseline and peak diameters were calculated. As the 95% CIs (1.0–1.3) and slopes (> 0.97) were all close to unity, allometric scaling of the FMD data was not performed in accordance with the expert guidelines (Atkinson et al. 2013).

### Statistical analysis

Normality was assessed using the Shapiro–Wilk test. If necessary, variables not normally distributed were log-transformed using the natural logarithm method. Outliers were detected and removed using mean ± 2 standard deviation (SD); 1 participant’s FMD_SRAUC_ datum was removed from IPC and data of 5 participants were removed from RIPC FMD_SRAUC_ analysis as outliers. All other data points for all other variables were not detected as outliers and thus included in the analysis. Jones et al. (2014) report FMD increased from 5.0 ± 2.2% to 6.6 ± 2.3% following repeated IPC, yielding an effect size of 0.71. Assuming a similar effect size in our study, a conservative correlation of 0.1 amongst repeated measures and nonsphericity corelation ε of 0.5, a two-way within subjects’ factor ANOVA would require a minimum sample size of 10 participants to yield a statistically significant difference at 5% alpha and 80% Power. Our baseline diameter and percentage FMD showed excellent test-retest reliability (intraclass correlation of 0.997, 95% confidence interval [CI: 0.982–0.999], *p* < 0.001; and 0.996, [95% CI: 0.978–0.999], *p* < 0.001; respectively). Values of coefficient of variation for method error were 1.01% and 2.73%, respectively.

Given all study participants did not undergo bilateral Radial artery imaging and data presented here are therefore essentially from two study groups (i.e., IPC assessments group (*n* = 11) and RIPC assessments group (*n* = 16), these groups were treated as separate cohorts. Therefore, a two-way within-subject factors repeated measures ANOVA was used to assess differences between intervention (either IPC or RIPC as appropriate), COX inhibition (pre- and post-aspirin), and interaction (i.e., Time x Treatment). Tukey’s post-hoc analysis was used where appropriate. ∆ change in FMD% pre- and post-aspirin before and after IPC / RIPC intervention was compared using paired *t*-test. Partial Eta-squared (*n*^2^p) values were calculated for repeated measures ANOVA and Cohen’s *d*z for the paired comparisons. A statistical significance was determined as *p* value of ≤ 0.05. Data are presented as mean ± SD unless stated otherwise. Statistical analyses were conducted using GraphPad Prism (version 10.5.0) and G*Power (version 3.1.9.6), as appropriate.

## Results

### Baseline characteristics

Overall, study participants were young individuals (24 [22–30] years, median [interquartile range]) with a BMI of 25 [24–29] Kg/m^2^ (Table 1). Average resting systolic and diastolic BP were within normal limits (118 ± 11 and 76 ± 8 mmHg, respectively, mean ± standard deviation: Table 1). Weekly alcohol consumption among most participants ranged from 0 to 4 units. Two 22 years old male participants reported consuming 12 and 20 units / week respectively. However, as their vascular data did not differ from the rest of the cohort, they were included in further analysis. Participants characteristics of individual IPC and RIPC groups are presented in Supplement File (Supplement Table 1 and Supplement Table 2).

### IPC intervention arm

**Fig. 1:**
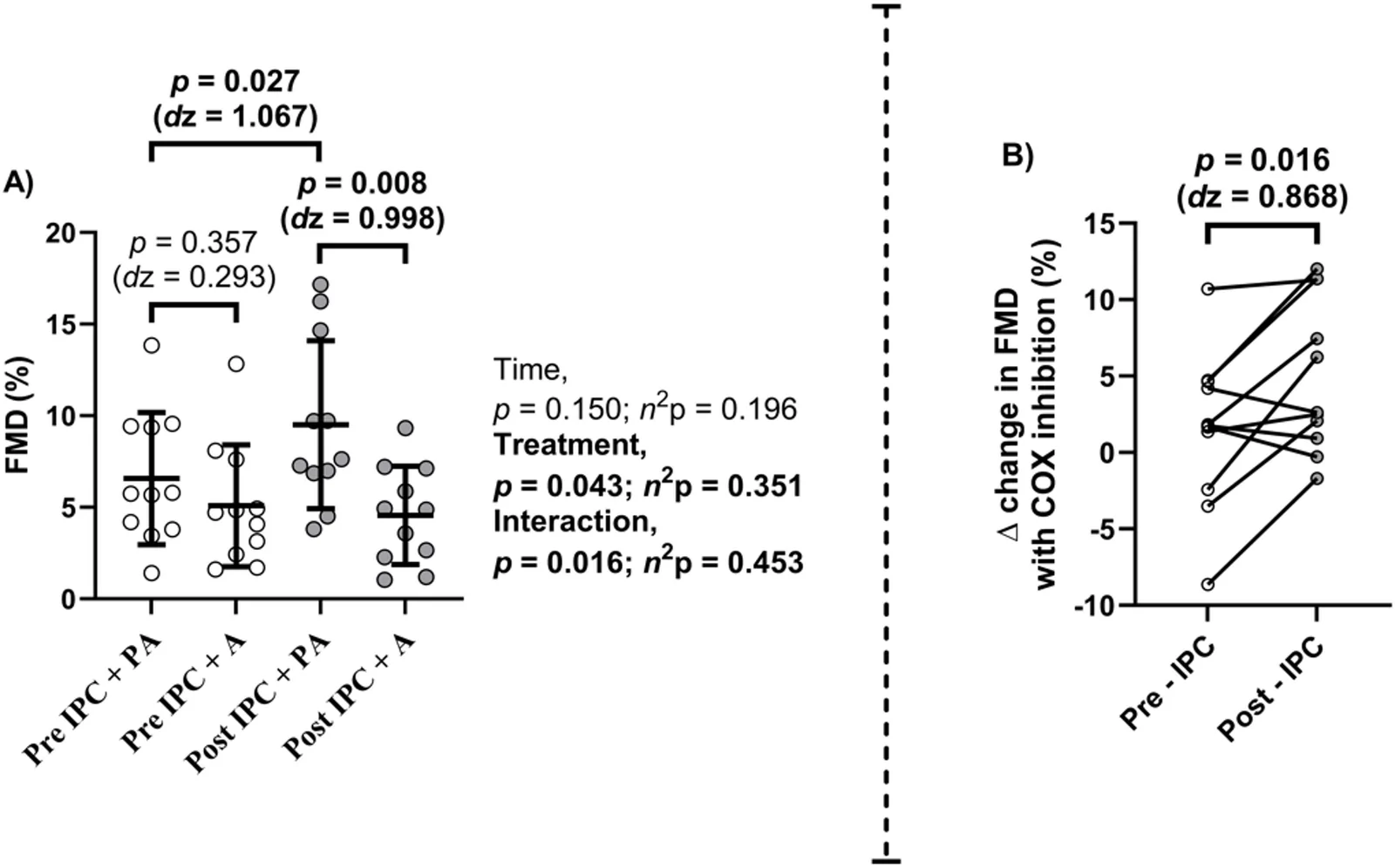
Radial artery FMD (%) from the IPC arm (**A**) and delta change in percentage FMD with COX inhibition pre and post IPC (**B**). For panel A, statistical comparisons made using a two-way within-subject factors repeated measures ANOVA with Tukey’s HSD used for post-hoc analysis. For panel B, statistical comparisons were made using paired *t*-test. Partial Eta-squared (*n*^2^p) and Cohen’s *d*z were calculated, as appropriate. For non-normally distributed data, log transformation was applied prior to statistical comparisons. Data are presented as mean ± SD. Abbreviations: ∆, delta change; A, aspirin; FMD, flow-mediated dilation; IPC, ischemic preconditioning; PA, pre-aspirin

Baseline arterial diameters remained unaltered between conditions (*p >* 0.05; Table 2). Peak diameter and SRauc were also not different between conditions (*p >* 0.05; Table 2). One-week of alternate day IPC treatment significantly increased pre-aspirin absolute FMD (*p* < 0.05, Table 2), percentage FMD (*p =* 0.027, Tukey’s; Fig. 1) and FMD_SRAUC_ (*p* = 0.004, Tukey’s; Table 2). Whilst Aspirin ingestion pre IPC failed to attenuate FMD responses; Aspirin use post IPC did significantly attenuate the absolute FMD, FMD % and FMD_SRAUC_ responses (*p <* 0.01, Tukey’s; Table 2; Fig. 1A). Paired-samples *t*-test revealed that ∆ change in FMD % before and after aspirin ingestion increased from 1.48 ± 5.05% pre IPC to 4.95 ± 4.96% post IPC (*p* = 0.016; *d*z = 0.868; Fig. 1B).

**Table 2:** Radial artery responses before and after aspirin ingestion in the ischemic preconditioning intervention arm

	Intervention IPC Arm (*n* = 11)			
		Pre-aspirin	Aspirin	P values (*n*^2^p)
Baseline Diameter (mm)	Pre IPC	2.44 ± 0.47	2.36 ± 0.44	Time: 0.168 (0.183) Treatment: 0.850 (0.003) Interaction: 0.118 (0.223)
	Post IPC	2.47 ± 0.50	2.54 ± 0.58	
Peak diameter (mm)	Pre IPC	2.59 ± 0.45	2.48 ± 0.47	Time: 0.094 (0.255) Treatment: 0.120 (0.224) Interaction: 0.377 (0.079)
	Post IPC	2.69 ± 0.48	2.65 ± 0.59	
Absolute FMD (mm)	Pre IPC	0.15 ± 0.07	0.12 ± 0.09	Time: 0.124 (0.220) Treatment: **0.036** (**0.371**) Interaction: **0.023** (**0.417**)
	Post IPC	0.22 ± 0.07*	0.11 ± 0.07†	
SR_AUC_ (x10^3^ s^− 1^)	Pre IPC	15.60 ± 5.10	15.42 ± 6.41	Time: 0.734 (0.008) Treatment: 0.443 (0.037) Interaction: 0.362 (0.085)
	Post IPC	17.45 ± 5.03	15.06 ± 5.84	
FMD_SRAUC_ (%)	Pre IPC	7.04 ± 3.64	5.23 ± 3.31	Time: **0.047** (**0.368**) Treatment: **0.021** (**0.464**) Interaction: **0.004** (**0.626**)
	Post IPC	11.41 ± 4.50*	4.76 ± 2.81†	

Radial artery responses before and after IPC and aspirin ingestion from the intervention arm. Each arm’s responses were assessed using a two-way within-subject factors repeated measures ANOVA for Time (i.e., pre IPC or post IPC, as appropriate), Treatment (Cox inhibition with aspirin) and interaction effects; post hoc analysis was performed using Tukey’s test﻿

*n*^*2*^*p* partial Eta-squared, *FMD* flow-mediated dilation, *FMD*_*SRAUC*_ shear rate area-under-the-curve as covariate corrected flow-mediated dilation, *IPC* ischemic preconditioning, *SR*_*AUC*_ shear rate area-under-the-curve

*p < 0.05 vs. Pre IPC + pre-aspirin, †*p* < 0.05 vs. Post IPC + pre-aspirin

Statistical significance was set at p < 0.05; significant results are presented in bold

### Contralateral RIPC arm

**Fig. 2:**
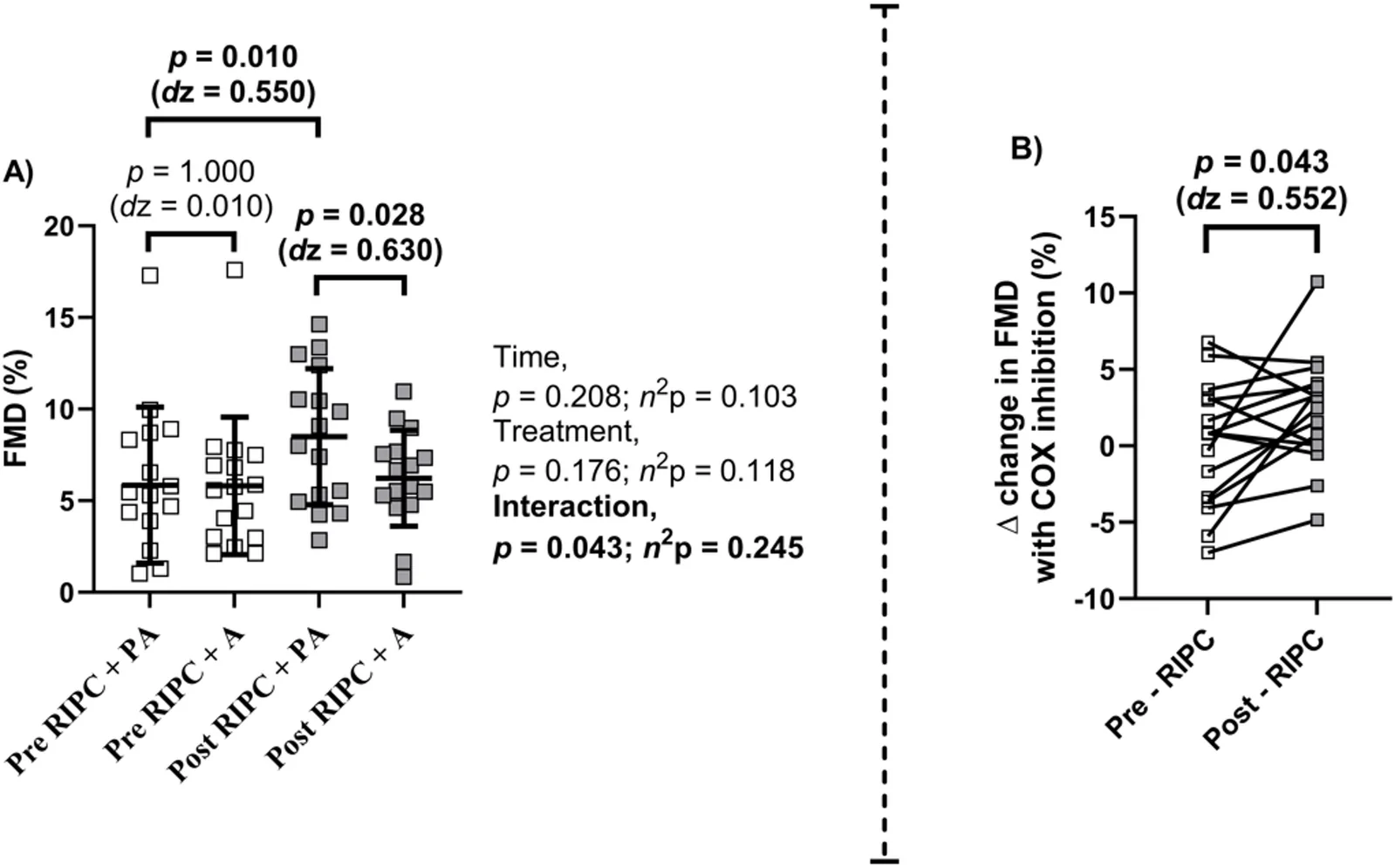
Radial artery FMD (%) from the RIPC arm (**A**) and delta change in percentage FMD with COX inhibition pre and post RIPC (**B**). For panel A, statistical comparisons made using a two-way within-subject factors repeated measures ANOVA with Tukey’s HSD used for post hoc analysis. For panel B, statistical comparisons were made using paired *t*-test. Partial Eta-squared (*n*^2^p) and Cohen’s *d*z were calculated, as appropriate. For non-normally distributed data, log transformation was applied prior to statistical comparisons. Data are presented as mean ± SD. Abbreviations: ∆, delta change; A, aspirin; FMD, flow-mediated dilation; PA, pre-aspirin; RIPC, remote ischemic preconditioning

In the contralateral arm, baseline diameters were also not different between the four conditions (Table 3). Aspirin use decreased the peak increase in diameter (*treatment effect*, *p* = 0.021; Table 3). A trend for increase in absolute FMD and FMD_SRAUC_ following RIPC was observed which appeared to be attenuated with aspirin (*interaction effect*, *p* < 0.083; Table 3). However, FMD % did increase significantly following RIPC treatment (*p* = 0.010, Tukey’s; Fig. 2) and aspirin ingestion post-RIPC attenuated this now increased FMD (*p* = 0.028, Tukey’s; Fig. 2A). Absolute difference in percentage FMD before and after aspirin ingestion increased from 0.04 ± 4.05% pre RIPC to 2.27 ± 3.60% post RIPC (*p* = 0.043; *d*z = 0.552; Fig. 2B).

**Table 3:** Radial artery responses before and after aspirin ingestion in the contralateral remote ischemic preconditioning arm

	Contralateral RIPC Arm (*n* = 16)			
		Pre-aspirin	Aspirin	P values (*n*^2^p)
Baseline Diameter (mm)	Pre RIPC	2.39 ± 0.35	2.35 ± 0.44	Time: 0.583 (0.021) Treatment: 0.074 (0.197) Interaction: 0.668 (0.013)
	Post RIPC	2.35 ± 0.49	2.28 ± 0.46	
Peak diameter (mm)	Pre RIPC	2.53 ± 0.38	2.48 ± 0.45	Time: 0.829 (0.003) Treatment: **0.021** (**0.308**) Interaction: 0.282 (0.077)
	Post RIPC	2.54 ± 0.51	2.42 ± 0.48	
Absolute FMD (mm)	Pre RIPC	0.14 ± 0.10	0.13 ± 0.08	Time: 0.204 (0.105) Treatment: 0.172 (0.121) Interaction: 0.069 (0.204)
	Post RIPC	0.19 ± 0.08	0.14 ± 0.06	
SR_AUC_ (x10^3^ s^− 1^)	Pre RIPC	13.59 ± 6.24	10.96 ± 4.36	Time: 0.995 (0.007) Treatment: **0.012** (**0.469**) Interaction: 0.415 (0.175)
	Post RIPC	14.59 ± 5.84	9.94 ± 4.36	
FMD_SRAUC_ (%)	Pre RIPC	7.51 ± 4.03	5.41 ± 4.39	Time: 0.455 (0.028) Treatment: **0.007** (**0.591**) Interaction: 0.083 (0.098)
	Post RIPC	8.89 ± 3.08	5.47 ± 2.49	

Radial artery responses before and after RIPC and aspirin ingestion from the contralateral arm. Each arm’s responses were assessed using a two-way within-subject factors repeated measures ANOVA for Time (i.e., pre RIPC or post RIPC, as appropriate), Treatment (Cox inhibition with aspirin) and interaction effects; post hoc analysis was performed using Tukey’s test

*n*^*2*^*p* partial Eta-squared, *FMD* flow-mediated dilation, *FMD*_*SRAUC*_ shear rate area-under-the-curve as covariate corrected flow-mediated dilation, *RIPC* remote ischemic preconditioning, *SR*_*AUC*_ shear rate area-under-the-curve

*p < 0.05 vs. Pre RIPC + pre-aspirin, †*p* < 0.05 vs. Post RIPC + pre-aspirin

Statistical significance was set at p < 0.05; significant results are presented in bold

## Discussion

Aim of this study was to investigate whether conduit artery endothelial improvements conferred with repeated IPC and RIPC and witnessed through improvements in FMD are, at least in part, mediated by COX-derived endothelial vasodilators. To our knowledge, this has not been investigated previously. In agreement with our hypothesis, we found that one week of repeated IPC significantly enhanced percentage FMD responses which were attenuated with COX inhibition (oral aspirin) only after the weeklong preconditioning treatment but not before it. Moreover, although we did observe similar responses for percentage FMD data in the contralateral RIPC arm, these changes were not consistent across all measures of FMD and therefore require further investigation. Collectively, these findings reveal an enhanced contribution of vasoactive prostanoids to the endothelial adaptations induced by repeated ischemic preconditioning.

Previous studies have shown that repeated IPC improves endothelium-dependent vasoreactivity not only at the site of ischemic stimulus but also in remote vascular territories. For example, Jones et al. (2014) reported that seven consecutive days of IPC significantly increased FMD in both the treated and contralateral RIPC arms, with effects persisting even eight days after termination of the intervention. Bond et al. (2024) expanded on this work by showing that six unilateral IPC sessions delivered over a two-week period produced equal bilateral FMD improvements. These IPC induced vascular adaptations have traditionally been attributed to enhanced eNOS activity and NO bioavailability (Chen et al. 2002; Rifkind et al. 2014; kimura et al. 2007). However, endothelium is a shared site of release for NO and PGI_2_, two potent synergistic vasodilators. Whilst previous studies have largely focused on demonstrating improvements in endothelial function with IPC and/or RIPC, or on exploring the role of eNOS pathways, present study is first of its kind to explore (and demonstrate) mechanistic contribution of COX-dependent prostanoid to improvements in endothelium dependent FMD which occurs with repeated IPC / RIPC treatments.

Herein, absolute FMD, percentage FMD and FMD_SRAUC_ significantly improved following the weeklong alternate day IPC protocol which was consistently attenuated after COX inhibition. One mechanism which can explain the observed findings possibly relates to the localised ischemic / hypoxic stimulus. In-vitro studies have demonstrated that hypoxia upregulates COX and PGI_2_ synthase expression in human vascular smooth muscle cells and human endothelial cells, resulting in increased production and activity of PGI₂ (Camacho et al. 2011). Exposure of human umbilical vein endothelial cells to hypoxic stimulus also upregulates COX-2 and PGI_2_ production without a concomitant rise in the production of vasoconstrictive thromboxane A₂ (Cook-Johnson et al. 2006). Moreover, localised ischemia of an arm, as would be observed with the IPC protocol, increases intravascular concentrations of adenosine within the ischemic tissue (Costa et al. 1999b), presence of which in vascular compartments has separately been shown to enhance PGI_2_ production (Nyberg et al. 2010; Mortensen et al. 2009). The ischemic / hypoxic impact of IPC is expected to encompass the entire arm and not just the intravascular compartment. Therefore, the contribution of extravascular tissue such as skeletal muscles cannot be ruled out. Indeed, when cultured endothelial cells are exposed to hypoxic myocytes (cardiac), this leads to an increased expression of endothelial COX-2 and parallel upregulation of PGI_2_ and prostaglandin E_2_ production (Wu et al. 2003). Although we are unaware of such data from skeletal myocytes, forearm skeletal muscle oxygen stores do substantially deplete within 5 min of ischemia (Hampson and Piantadosi 1988; Hamaoka et al. 2000; Perlet et al. 2024). Moreover, exercise induced production of PGI_2_ which has previously been linked to muscle oxygen uptake (Zoladz et al. 2009) attenuates with oxygen supplementation (Junejo et al. 2020). Therefore, the localised hypoxic / ischemic stimulus of the IPC treatment may be a direct stimulus for increased activity and/or production of COX derived endothelium-dependent vasodilators.

Another mechanism which helps explain the findings relates to the circulating concentrations of vasoactive substances. For example, IPC increases circulating concentrations of vascular endothelial growth factor (VEGF) and endothelial progenitor cells (kimura et al. 2007), thereby contributing to the localised and systemic improvements in endothelial health and function (Hill et al. 2003). Moreover, IPC increases circulating concentrations of brain derived neurotrophic factor (BDNF); BDNF and VEGF potentiate endothelial PGI_2_ production (He et al. 1999; Nakahasi et al. 2000; Rytter et al. 2020). Additionally, whilst the release of reactive oxygen species (ROS) is triggered by the initial ischemia-reperfusion stimulus, the repeated low level ROS activity of IPC treatment triggers an increase in the natural anti-oxidant defence mechanisms (He et al. 2013; Quarrie et al. 2012) which perhaps increases the bioavailability of endothelial PGI_2_. These circulating influences are unlikely to be confined to the IPC limb and must contribute to the systemic improvements in the endothelial health and function via RIPC. Indeed, Rytter et al. (2020) have observed improved microvascular endothelial health and potentiation of PGI_2_ release following even an acute RIPC treatment. Similarly, Oxman et al. (1997) have also previously recorded an increase in the PGI_2_ concentrations in coronary effluent following RIPC. Finally, upregulation of COX-dependent mediators such as PGI₂ also have an important role in the inhibition of platelet aggregation and reducing leukocyte adhesion (Moncada and Vane 1981). These signals likely act in concert to enhance local and remote endothelial repair / function with IPC.

Importantly, findings of our study should also be interpreted within the broader literature around impact of IPC / RIPC on shear stress-induced endothelial mechanotransduction and NO bioavailability via eNOS activation (kimura et al. 2007; Rifkind et al. 2014). IPC increases eNOS contributions through a complex signalling cascade involving phosphorylation and protein translocation. In the early phases, repeated cycles of ischemia and reperfusion trigger the release of adenosine and bradykinin which activate the phosphatidylinositol 3-kinase / protein kinase B (PI3K/Akt) and protein kinase A (PKA) pathways (Yang et al. 2013; Cohen et al. 2000; Costa et al. 1999a). These kinases phosphorylate eNOS thereby increasing NO production (Yang et al. 2013). IPC also disrupts the inhibitory interaction between eNOS and scaffolding protein caveolin-1, facilitating enzyme’s translocation to cytosol / plasma membrane for vasodilation (Su 2014; Reiner et al. 2001; Koneru et al. 2007). It is worth remembering that the vascular effects of eNOS activity are also mediated by a complex crosstalk between cyclic nucleotides (i.e., guanosine monophosphate (cGMP) and adenosine monophosphate (cAMP)) and phosphodiesterases (PDEs); NO production by eNOS increases cGMP levels which also modulates cAMP concentrations through specific PDEs (Lorigo et al. 2021; Pavlaki and Nikolaev 2018; Aizawa et al. 2003; Zaccolo and Movsesian 2007). cAMP is the primary second messenger involved in PGI_2_ mediated vasodilation (Kukovetz et al. 1979). This raises two considerations. Firstly, whilst the results of prior studies which have utilised eNOS inhibition to determine NO dependent contribution to vasodilation which follows IPC (kimura et al. 2007), may not only be revealing reduced production / activity of cGMP but also of secondary indirect attenuation of cAMP activity. Secondly, with relation to the results presented herein, it is also possible that the increased activity of eNOS and cGMP overtime with repeated IPC / RIPC has enhanced the contributions of COX-dependent vasodilators to FMD by aiding a sustained increase in cAMP levels. Therefore, it is likely that the findings presented herein are a reflection of IPC / RIPC’s impact on cGMP and cAMP pathways and do not suggest exclusiveness. Nonetheless, future studies are required where cGMP and cAMP concentrations are assessed in vascular / endothelial cells, and eNOS and COX pathways are serially inhibited before and after IPC / RIPC.

### Limitations and considerations

The findings from this study should be interpreted in light of the following considerations. Although we planned for all participants to have their FMD responses recorded from IPC and RIPC arms, technical limitations and limited time availability from certain participants prevented this. Separately, the study did not obtain blood samples from participants. As a result, we were unable to directly quantify plasma concentrations of different prostanoids. The precise mechanisms by which IPC and RIPC enhance the contribution of COX-dependent vasodilators remained beyond the scope of this study, and we can therefore only speculate. Young healthy participants were recruited in the study to determine whether repeated IPC / RIPC induced improvements in endothelial function are at least in part mediated by COX pathways. Choice of the study cohort was aimed at avoiding preexisting attenuation in endothelial function associated with aging and comorbidities, thus providing a clearer mechanistic insight in healthier controlled physiological conditions. Future studies should however expand this work into cohorts with known endothelial dysfunction, such as older individuals and individuals with known cardiovascular conditions. Endothelium-independent vasodilation was not assessed herein. IPC and RIPC exhibit a dual phase preconditioning protection (Loukogeorgakis et al. 2005). This study assessed the impact of repeated IPC treatments on conduit artery FMD twenty-four hours after final preconditioning ischemia. Therefore, we cannot comment on the influence of IPC on COX-dependent pathways and associated endothelial function before or beyond this timepoint. Further, although a significant attenuation in percentage FMD was observed with aspirin in the contralateral arm following RIPC treatment along with an improvement in the contribution of COX-pathway to FMD responses (Fig. 2B), these findings were not consistent across all measures of FMD (Table 3). Perhaps the magnitude / intensity of COX-dependent endothelial adaptations in remote tissue are lower compared to those witnessed with localized IPC. Therefore, it is plausible that a longer duration and/or higher frequency of IPC exposure is required for robust COX-dependent effect in remote vascular territories. However, this requires a further independent assessment. Finally, improvements in COX-dependent endothelial function with IPC / RIPC may have broader implications for vascular protection, including endothelial repair and anti-thrombotic function (Katusic et al. 2012; Higgs et al. 1997). The results presented herein do indicate important translational potential but further assessments in vulnerable cohorts are required and these questions were not the remit of this particular study. Given that the present study was conducted in a relatively young and healthy cohort under controlled conditions, the extent to which these findings generalise to older adults who are prone to endothelial dysfunction but may also exhibit attenuated COX-dependent conduit artery hyperaemia (Schrage et al. 2007), further investigations are warranted. Similarly, generalisability of these results to clinical cohorts and influence of sex hormones on the observed results remains to be determined.

## Conclusion

The main finding of this study is that the contribution of cyclooxygenase derived endothelium dependent vasodilators to flow mediated dilation significantly increases with one week of alternate day repeated ischemic preconditioning. This suggests that cyclooxygenase dependent vasodilator prostanoids contribute to improvements in vasomotor function observed with repeated ischemic preconditioning. Impact of remote ischemic preconditioning however requires further investigation.

## Supplementary Information

Below is the link to the electronic supplementary material.

Supplementary Material 1

## Data Availability

Data are available from the corresponding author to verified researchers upon request.

## Abbreviations

BMI: Body mass index
COX: Cyclooxygenase
FMD: Flow mediated dilation
IPC: Ischemic preconditioning
L-NMMA: N^G^-monomethyl-L-arginine
NO: Nitric oxide
PGI_2_: Prostacyclin/prostaglandin I_2_
RIPC: Remote ischemic preconditioning
SR_AUC_: Shear rate area under the curve
