# Supplementary material for: Cyclooxygenase-dependent contribution to increased flow mediated dilatation following a week of repeated ischemic preconditioning
Source: Eur J Appl Physiol. 2026 Jun 29;126(10):5281–91. doi: 10.1007/s00421-026-06324-4 (PMC13633948; doi:10.1007/s00421-026-06324-4)
Supplement: Supplementary file 1 [file 421_2026_6324_MOESM1_ESM.docx]

**Supplementary Table 1.** Characteristics for participants undergoing assessment of the ischemic preconditioning intervention arm.

| **Characteristics** | |
| --- | --- |
| Age, years | 24 [22 – 30] |
| Sex, *m*/*f* | 7/4 |
| Height, cm | 177 ± 9 |
| Weight, Kg | 82 ± 10 |
| BMI, Kg/m^2^ | 26 ± 4 |
| Systolic blood pressure, mmHg | 121 ± 10 |
| Diastolic blood pressure, mmHg | 75 ± 8 |
| Mean arterial blood pressure, mmHg | 94 ± 9 |
| Pulse pressure, mmHg | 46 ± 7 |
| Alcohol use (*n*) | 6 |
| Multivitamins use (*n*) | 3 |
| Iron use (*n*) | 0 |
| Fish Oil Use (*n*) | 0 |

Descriptive characteristics of the participants in the study who completed assessment of the intervention arm exposed to the cuff stimulus (*n* = 11). Continuous variables are presented as mean ± SD for normal distributed data and median [IQR] for non-normal distributed data. Discrete quantitative data are presented as count. Abbreviations: BMI, body mass index.

**Supplementary Table 2.** Characteristics for participants undergoing assessment of the remote ischemic preconditioning contralateral arm

| **Characteristics** | |
| --- | --- |
| Age, years | 23 [21 – 28] |
| Sex, *m*/*f* | 10/6 |
| Height, cm | 174 ± 9 |
| Weight, Kg | 81 ± 22 |
| BMI, Kg/m^2^ | 27 ± 6 |
| Systolic blood pressure, mmHg | 119 ± 12 |
| Diastolic blood pressure, mmHg | 77 ± 7 |
| Mean arterial blood pressure, mmHg | 94 ± 8 |
| Pulse pressure, mmHg | 41 ± 12 |
| Alcohol use (*n*) | 6 |
| Multivitamins use (*n*) | 4 |
| Iron use (*n*) | 1 |
| Fish Oil Use (*n*) | 2 |

Descriptive characteristics of the participants in the study who completed assessment of the contralateral arm not exposed to the cuff stimulus (*n* = 16). Continuous variables are presented as mean ± SD for normal distributed data and median [IQR] for non-normal distributed data. Discrete quantitative data are presented as count. Abbreviations: BMI, body mass index.
